# SEXUAL ACTIVITY OF OLDER ADULTS: WE’RE ASKING THE WRONG QUESTIONS

**DOI:** 10.1093/geroni/igac059.1920

**Published:** 2022-12-20

**Authors:** Janie Steckenrider

**Affiliations:** Loyola Marymount University, Los Angeles, California, United States

## Abstract

Sexual activity of older adults is an under researched area as most surveys on sexual behavior end at age 60 reflecting the myth that older adults are not sexually active. Only recently has survey data asked specifically about sexual activity of those ages 60 to 95. Their consensus is over half of males and a third of females over 70 are sexually active. Most striking is the current body of research almost exclusively defines sexual activity measured as partnered sexual behaviors of intercourse, fondling, kissing, touching. Given the reality for many older adults lacking an active sex partner due to death, sexual dysfunction, or serious illness, the aim of this study was to determine if the right survey questions are being asked for older adults. Seven major surveys, underlying most current research, were analyzed regarding solitary sex (masturbation) compared to partnered sex. Results of this study found extensive questioning about aspects of partnered sex including pleasure, satisfaction, pain, relationship status, sexual functioning, anxiety, individual sexual acts, etc. compared to only two questions about masturbation, both only about frequency. The psychological and physiological benefits of sexual activity, both partnered and solo sex, are well documented and correlate with higher life satisfaction for older adults. Sexual activity needs to be redefined to also include solitary sex and this begins by asking the right questions. This study has implications for the need to bring a broader perspective in promoting a healthy sex life among older adults, defined both as partnered and solitary sex.

